# Scar Remodeling with the Association of Monopolar Capacitive Radiofrequency, Electric Stimulation, and Negative Pressure

**DOI:** 10.1089/pho.2016.4180

**Published:** 2017-05-01

**Authors:** Giovanni Nicoletti, Paola Perugini, Sara Bellino, Priscilla Capra, Alberto Malovini, Omar Jaber, Marco Tresoldi, Angela Faga

**Affiliations:** ^1^ Plastic and Reconstructive Surgery, Department of Clinical Surgical Diagnostic and Pediatric Sciences, University of Pavia, Pavia, Italy.; ^2^ Advanced Technologies for Regenerative Medicine and Inductive Surgery Research Center, University of Pavia, Pavia, Italy.; ^3^ Plastic and Reconstructive Surgery Unit, Istituti Clinici Scientifici Maugeri, Pavia, Italy.; ^4^ Department of Drug Sciences, University of Pavia, Pavia, Italy.; ^5^ Laboratory of Informatics and Systems Engineering for Clinical Research, Istituti Clinici Scientifici Maugeri, Pavia, Italy.; ^6^ Freelance Plastic Surgeon, San Martino Siccomario, Pavia, Italy.

**Keywords:** scar, radiofrequency, negative pressure, electrical stimulation therapy

## Abstract

***Objective:*** A study was established to objectively assess the effects of low-intensity electromagnetic and electric stimulation plus negative pressure on mature scars. ***Background:*** Radiofrequency plus negative pressure therapy demonstrated a favorable reorganization and regeneration of the collagen and elastic fibers and was proposed for the treatment of cellulitis and skin stretch marks. ***Methods:*** Twenty-six mature scars in 20 Caucasian patients (15 females and 5 males) were enrolled in the study. The treatments were carried out with a Class I, BF-type electromedical device equipped with a radiofrequency generator, an electric pulse generator, and a vacuum pump twice a week for 3 months. Corneometry, transepidermal water loss, elastometry, colorimetry, and three-dimensional skin surface pattern were objectively assessed with Multi Probe Adapter System MPA and PRIMOS pico. A subjective assessment was carried out with the VAS and PSAS scales. Each scar was compared before and after the treatment and with the skin in the corresponding healthy contralateral anatomical area at the same times. ***Results:*** Reduction of the scar surface wrinkling and overall scar flattening were demonstrated after the treatment. The scar slightly tended to approach the color and elasticity of healthy skin too. ***Conclusions:*** The combined local treatment of mature scars with low-intensity electromagnetic and electric stimulation in association with negative pressure might suggest a favorable synergic effect on the scar collagen and elastic fiber remodeling.

## Introduction

Scar formation is the ultimate outcome of wound repair in humans that takes place as a cascade consisting of overlapping inflammatory, proliferative, and remodeling phases. When the process of wound healing is uneventful after completion of the remodeling phase, the scar enters the so-called mature state according to the scheme proposed by the International Advisory Panel on Scar Management.^1^ Scar has no epidermal appendages and displays a collagen pattern of densely packed fibers. The tensile strength of wounded skin at best reaches only approximately that of unwounded skin.^2^ In addition, scar is brittle and less elastic than normal skin, although the regeneration of elastic fibers in the scar is still debated.^3^ In addition, scars are usually hypopigmented after full maturation even if they can become hyperpigmented in dark pigmented individuals or in lighter pigmented ones after exposure to UV radiation. In conclusion, the scar itself does not reproduce the features of normal skin, and therefore, it is still an unsolved functional and cosmetic issue despite the large number of treatment proposals: surgery, silicone gel sheeting, injected corticosteroids, pressure therapy, radiotherapy, laser therapy, cryotherapy, adhesive microporous hypoallergenic paper tape, and a number of miscellaneous therapies based on an anecdotal basis.^1^

A large number of literature reports demonstrate the effectiveness of radiofrequencies on favorable collagen remodeling through both immediate ultrastructural changes in the fibrils architecture and subsequent induced regeneration of new collagen and elastic fiber network.^4,5^ Therefore, radiofrequencies have been supposed to have favorable effects on scar remodeling, although such a correlation is actually controversial as suggested by a large number of literature reports that failed to prove any sound evidence.^6,7^

The association of radiofrequency, electric stimulation, and negative pressure has been reported effective for the treatment of cellulitis and skin stretch marks^8–11^ through the reorganization and regeneration of the collagen and elastic fibers. On that basis, the authors established a study to objectively assess these combined effects on mature scars in a human sample.

## Materials and Methods

A prospective controlled open clinical pilot trial was carried at the Advanced Technologies for Regenerative Medicine and Inductive Surgery Research Centre, University of Pavia, Pavia, Italy, in cooperation with the Plastic Surgery Unit, Department of Clinical Surgical, Diagnostic, and Pediatric Sciences and the Department of Drug Sciences of the University of Pavia, Italy.

The study was approved by the University of Pavia Ethical Committee on September the 19^th^, 2013.

The inclusion criteria were as follows: ages between 18 and 65 years and body mass index between 15 and 35. Exclusion criteria were as follows:

• Pacemaker implant
• Current or past 5 years history of chemotherapy
• Local sensation disturbances
• Epilepsy
• Local skin inflammation
• Local vascular diseases (thrombosis, thrombophlebitis, varicose veins)
• Difficult to heal wounds
• Anticoagulation therapy
• Severe renal failure
• Current or past 5 years history of anorexia or bulimia
• Current pregnancy or breastfeeding
• Patients with very poor skin compactness and elasticity requiring surgical treatment (mastopexy, abdominoplasty, brachioplasty, and so on)
• Thyroid diseases
• Adrenal diseases
• Current or recent past history of oral contraception
• Adult acne
• Recent development of hypertrichosis
• Menstrual cycle alterations
• History of frequent and massive body weight changes
• Current hypocaloric diet
• Past 6 months history of hormone regulating therapy
• Current pharmacological treatments of any type
• Use of any topical scar treatment within 3 months before the enrollment.

According to the former criteria, a homogeneous sample of 19 consecutive Caucasian patients (14 females and 5 males, 12 subjects Fitzpatrick photo-type 2 and 7 subjects Fitzpatrick photo-type 3) with an overall of 25 mature scars was enrolled in the study. Mean age was 31 years (minimum 21, maximum 58, median 24). The trial was carried out over a period of 17 months, from October 2013 to February 2015.

The scar was considered the experimental unit of the study, irrespective of the number of scars per patient. Multiple scars per patient were calculated as a single measurement value corresponding to the mean value of multiple measures. All of the scars had a history longer than 1 year (range 1–23, mean 8, median 7) and were considered mature and not hypertrophic according to the International Advisory Panel on Scar Management.^1^ Twenty scars were the outcome of a primary intention wound healing process and 6 followed a secondary intention wound healing (Table 1).

**Table 1.** The Scars Sample

*Scar*	*Wound healing*	*Aethiology*	*Site*	*Size (mm)*
1	Primary	Abdominoplasty	Lower abdomen	200 × 15
2	Primary	Abdominoplasty	Lower abdomen	260 × 10
3	Primary	Appendicectomy	Right iliac fossa	26 × 6
4	Primary	Appendicectomy	Right iliac fossa	70 × 20
5	Primary	Appendicectomy	Right iliac fossa	40 × 10
6	Primary	Arthroplasty	Left knee	100 × 10
7	Primary	Fracture ORIF	Left elbow	150 × 10
8	Primary	Fracture ORIF	Left arm	210 × 15
9	Primary	Fracture ORIF	Right arm	140 × 10
10	Primary	Mole excision	Back	31 × 12
11	Primary	Mole excision	Right upper abdomen	25 × 10
12	Primary	Mole excision	Right arm	110 × 15
13	Primary	Hysterectomy	Lower abdomen	160 × 3
14	Primary	Trauma	Left knee	13 × 4
15	Primary	Trauma	Left iliac fossa	75 × 20
16	Primary	Cardiac surgery	Sternum	185 × 5
17	Primary	Arthroplasty	Left knee	35 × 9
18	Primary	Male genital surgery	Left iliac fossa	50 × 5
19	Primary	Vascular malformation excision	Left thigh	240 × 10
20	Secondary	Trauma	Left scapula	120 × 20
21	Secondary	Trauma	Left knee	40 × 8
22	Secondary	Burn	Left neck	60 × 60
23	Secondary	Burn	Left leg	80 × 80
24	Secondary	Burn	Left neck	86 × 50
25	Secondary	Trauma	Left arm	30 × 30

ORIF, open reduction and internal fixation.

### The equipment

The treatments were carried out with a Class I, BF-type electromedical device patented for noninvasive cosmetic applications called BiOne^®^ (Expo Italia Srl, Firenze, Italy).

It is equipped with a radiofrequency generator, an electric pulse generator, and a vacuum pump.

The radiofrequency generator emits a shielded capacitive variable radiofrequency signal (frequency range 0.5–1 MHz ±10%, maximum power 6 W at 500 Ohm, temperature output range 39°C–40°C). A biofeedback system allows for an automatic output frequency adjustment according to the individual patient's skin biological features.

The electric pulse generator emits a 5 Hz square wave with adjustable output up to 0.36 mA at 500 Ohm.

The two generators are mechanically, galvanically, and optically isolated.

The vacuum pump provides an adjustable negative pressure up to max 0.35 atm.

**FIG. 1.**
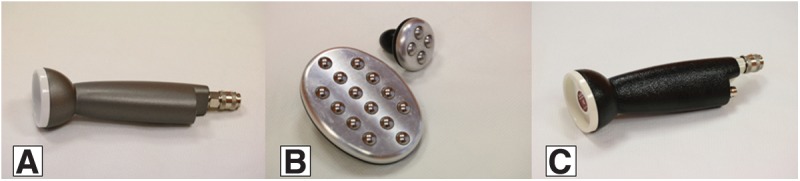
The device probes interacting with the human body: **(A)** the peeling probe connected to the vacuum pump; **(B)** the stimulating plates; and **(C)** the active probe.

The device is provided with a number of probes interacting with the human body (Fig. 1).

• The peeling probe is a resin bodied handle with a polyvinyl chloride (PVC) head covered with single use abrasive disks (diameter 150 mm, grain S1000) connected to the vacuum pump providing a negative pressure up to max 0.35 atm.
• The stimulating probe, connected to the electric pulse generator, are polyvinyl nitrate bodied handles, provided with an Anticorodal (Aluminum alloy) 110 plate supporting a set of AISI 316 (austenitic stainless steel alloy) spheres (14 spheres for the large probe and 4 spheres for the small one).
• The active probe is a resin-bodied handle provided with a white PVC disk connected to the same vacuum pump as the peeling probe, centered by a red epoxy glass core connected to the radiofrequency generator. Both the electric square wave and the radiofrequency are returned back to their generators through a neutral electrode, consisting in an Aluminum cylinder, held in the palm of the patient's hand.

### The procedure

Each treatment takes place in four steps.

First step: a soft peeling with the peeling probe is performed with a mechanical gommage implemented by the negative pressure; it lasts a few minutes, until the skin surface turns light red.

Second step: electric stimulation with the stimulating probes; it lasts from 2 to 6 min; the intensity of the square wave is regulated by the operator according to the patient's sensory threshold.

Third step: activation with the active probes; it lasts 10 min starting with the vacuum pump and then switching to the radiofrequency generator as a slight skin erythema shows.

Fourth step: stimulation of the lymphatic drainage by moving the active probe switched on radiofrequency mode along the course of the lymphatic vessels.

To enhance the thermal and electrical contact between the treatment tip and the skin, in the second, third, and fourth steps, a water gel containing Hyaluronic acid and plant extracts acting as a conductive medium is applied on the skin surface.^12,13^

### Assessments

The objective assessments were carried out at the Laboratory of Pharmaceutical Chemistry of the Department of Drug Sciences at the University of Pavia, Italy, using two instrumental devices:

1. Multi Probe Adapter System MPA (Courage and Khazaka, Koln, Germany) equipped with Cutometer MPA 580, Corneometer CM825, Tewameter TM300, Mexameter MX18, and Colorimeter CL 400 allows assessment of skin corneometry, transepidermal water loss (TEWL), elastometry, and colorimetry.
2. PRIMOS pico (GFMesstechnik; GmbH, Teltow, Germany) allows a three-dimensional (3D) skin scan.

These diagnostic techniques already proved their effectiveness in the objective anatomical functional assessment of the skin in several previous reports in the literature.^14–19^

All of the devices are CE certified and passed the safety tests before use.

The anatomical functional parameters under study in our sample of scars were corneometry, TEWL, elastometry, colorimetry, and 3D skin surface pattern.

As the sample included scars with different size, the smallest was considered as the unit of measurement and the assessments were carried out in the midpoint of this unit. The larger sized scars were approximately divided in segments corresponding to the unit of measurement, and the arithmetical average from all the units' values was assumed as the value for the whole scar.

#### Corneometry

As skin is a dielectric medium, all variations in hydration correspond to changes in the skin capacity. The hydration of the stratum corneum was assessed with a 49 mm^2^ surface probe allowing precise measurement in 1 sec within a 10–20 μm depth range.

#### TEWL

TEWL was assessed in terms of gr/m^2^/h by a skin evaporimeter made of a small cylindrical open chamber (1 cm in diameter, 2 cm in height) with a couple of hygrometric sensors connecting to a microprocessor plugged into a computer workstation. The device allows recording of the TEWL (ranging from 0 to 90 g/m^2^/h), the relative humidity (ranging from 0% to 100%), and the probe temperature.

#### Elastometry

The cutaneous elasticity was assessed through a Cutometer measuring the vertical deformation of the skin induced by vacuum aspiration. A negative pressure of 450 mbar was applied on the skin for a time of 1–3 sec through a 2 mm diameter probe. Each aspiration is followed by a release time, allowing the skin to return to its resting condition. The probe is provided with an optic sensor assessing variation of light transmission due to the aspirated skin bulking inside the probe.

The following parameters have been considered reliable indicators of the skin elasticity:

• Skin compactness (R0): the passive skin behavior following application of negative pressure
• Skin resistance (R2): the resistance against the return to the rest conditions at the end of suction
• Net skin elasticity (R5): the ratio between the maximum skin extension and the residual skin deformity.
• The parameters were expressed on an arbitrary score scale.

#### Colorimetry

Skin colorimetry was measured using two methods: Mexameter and Colorimeter.

In the Mexameter method, a 5 mm diameter probe emits light at three different wavelengths (568, 660, and 870 nm). An optic sensor measures such a light after reflection on the skin. The device measures the emitted light absorption rate by both the melanin and hemoglobin, providing an arbitrary melanin index (MI) and an arbitrary hemoglobin index, respectively, range 0–999.

In the Colorimeter method, an 8 mm diameter probe emits white LED light. An optic sensor measures the light after reflection on the skin using an arbitrary score scale for the following parameters:

• Luminosity (L): range 0 (black)–100 (white)
• Green and red (A): tolerance −120/+120
• Blue and yellow (B): tolerance −120/+120

The skin color is calculated using the formula: L × A × B = ITA

#### 3D skin scan

The PRIMOS (Phaseshift Rapid *In vivo* Measurement of the Skin) system provides high-resolution assessment of skin surfaces by using phase-shifted light stripes projected by micromirrors to generate a 3D profile (area 18 × 13 mm). The reflected light is captured by a high-resolution camera, and a software package converts the image into a color-coded picture, with different colors for different heights. Skin reliefs and hollows are measured as follows:

• Maximum absolute height in μm of the skin profile calculated from the maximum depth of the skin hollows to the top of the skin reliefs
• Mean furrows depth (μm)
• Mean depth of the deepest furrow (μm)
• Maximum depth of the deepest furrow (μm)
• Furrow count
• Furrow overall volume (mm^3^)
• Overall furrow surface (mm^2^)
• Furrow surface ratio: percentage of the skin area with furrows versus the area without furrows
• Furrow overall length (mm): length sum of all furrows.

The 3D synthetic assessment of the skin surface was expressed by the function integral of the skin surface profile (Ra) and by the difference between the highest skin surface spot and the deepest skin furrow in μm (Rmax).

The subjective assessments were carried out using the VAS and PSAS scales.

Two separate VAS Scales (score range: 1–10) assessing the overall subjective perception of the scar were blindly submitted both to the patients and to a single dedicated medical researcher. The patients were also given a PSAS Scale (score range: 1–10) assessing the scar-related pain, color, stiffness, and thickness.

### Trial planning

A complete treatment cycle had a duration of 3 months. Each patient enrolled in the study underwent a preliminary consultation (t_0_) for the pretreatment baseline assessments. Then, 24 sequential treatment sessions with a 2 weekly schedule followed (t_1_–t_24_). Each treatment lasted 30 min. The effects of the sequential treatments were assessed at the time of the final consultation (t_25_), 3–13 (median 5) days after the last application.

A formal informed written consent for both the procedure and medical photography was obtained from all of the patients, and the study conformed to the Declaration of Helsinki.

The patients filled the VAS and PSAS questionnaires at the time of enrollment (t_0_), after 2 months of treatment (t_16_), and at the end of the treatment (t_25_).

The scars were assessed at the beginning and at the end of the study (t_0_ and t_25_), and a comparison was carried out between the measurements at these times. Patients were advised to not apply any topical moisturizing ointments 24 h before the measurements.

In addition, each scar was compared with the skin in the corresponding healthy contralateral anatomical area at the same times, to exclude all of the changes in the scars that might not be related to the treatment, thus providing an intrapatient control.

All of the collected data were gathered into a patient's comprehensive individual chart.

### Statistical methods

Individual-level measurements were calculated as the mean value of multiple measures for each individual patient (if >1 measurement was available) or by a single measurement otherwise. Quantitative variables distribution is described by median [25^th^, 75^th^ percentiles or interquartile range (IQR)]. The presence of statistically significant variations in terms of quantitative variables distribution between repeated measurements was performed by the *t*-test for paired samples or by the Wilcoxon test for paired samples when variables deviated from the normal distribution (Shapiro–Wilk test *p* value <0.05). The presence of statistically significant differences in terms of quantitative variables distribution between scar and the contralateral corresponding healthy skin was assessed by the *t*-test for unpaired samples or by the Wilcoxon rank-sum test for unpaired samples when variables deviated from the normal distribution (Shapiro–Wilk test *p* value <0.05). The correlation between individual-level variations observed by comparing before (t_0_) versus after the treatment (t_25_) measurements and age at scar was evaluated by the Pearson or Spearman correlation tests as appropriate. The presence of statistically significant differences in terms of individual-level variations observed by comparing before (t_0_) versus after the treatment (t_25_) measurements among aethiology classes was assessed by the one-way ANOVA test or by the Kruskal–Wallis test as appropriate. *p* values <0.05 were considered statistically significant. All statistical tests were performed by the R statistical software version 3.2.2 (www.r-project.org).

## Results

**FIG. 2.**
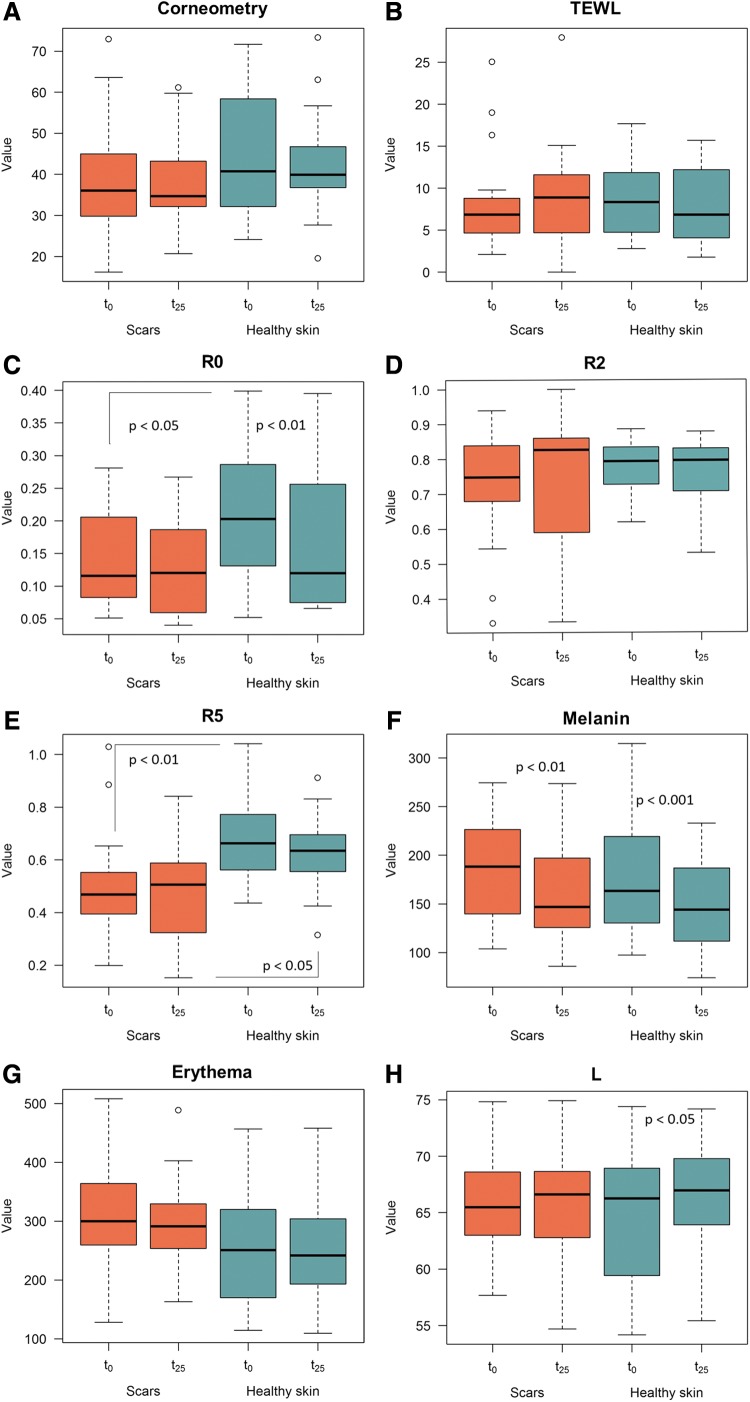

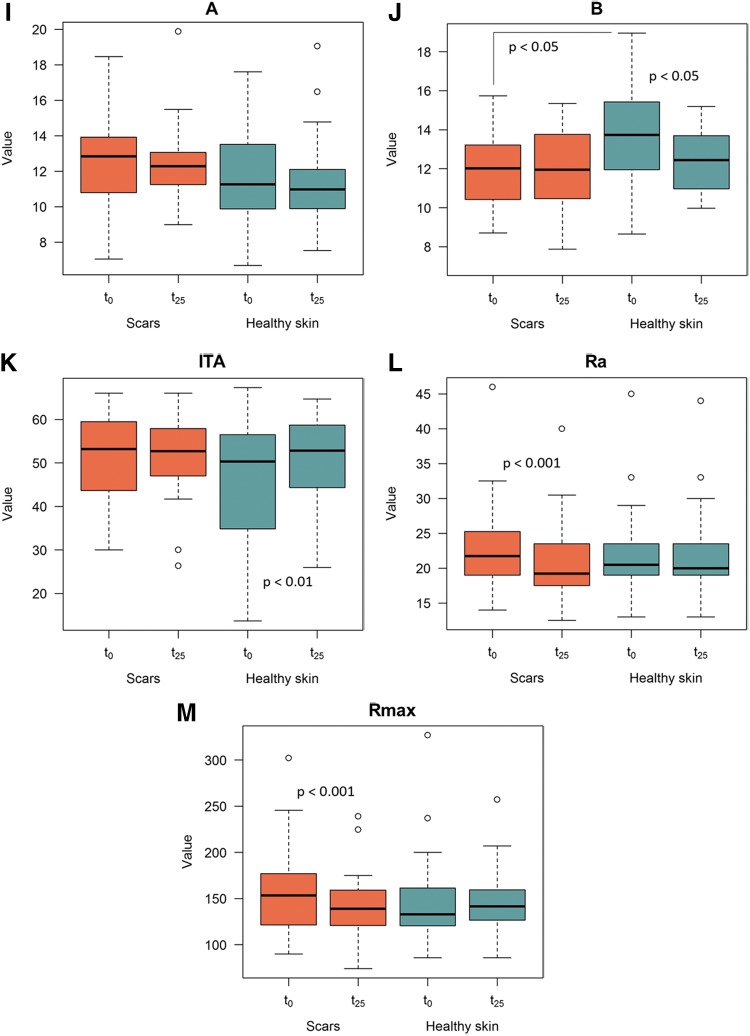
The parameters distribution: **(A)** Corneometry; **(B)** TEWL; **(C)** R0; **(D)** R2; **(E)** R5; **(F)** Melanin; **(G)** Erythema; **(H)** L; **(I)** A; **(J)** B; **(K)** ITA; **(L)** Ra; and **(M)** Rmax. Each boxplot graphically represents (from bottom to top) the lower nonoutliers limit, the 25^th^ percentile, the 50^th^ percentile (median value), the 75^th^ percentile, and the upper nonoutliers limit of each parameter's distribution at t_0_ and t_25_ for scars and healthy skin, respectively. Each dot represents an outlier measurement with respect to the corresponding distribution. TEWL, transepidermal water loss.

The results from the objective instrumental assessments are summarized in Table 2 and Fig. 2 (*a* to *m*).

**Table 2.** Variables Distribution at t_0_ and t_25_ and Corresponding Comparisons

	*St_0_*	*St_25_*	*HCSt_0_*	*HCSt_25_*	*St_0_ vs. St_25_*	*HCSt_0_ vs. HCSt_25_*	*St_0_ vs. HCSt_0_*	*St_25_ vs. HCSt_25_*
*Variable*	*Median (IQR)*	*Median (IQR)*	*Median (IQR)*	*Median (IQR)*	p	p	p	p
Corneometry	36.09 (30.03, 43.11)	34.67 (32.2, 42.77)	40.73 (32.2, 57.72)	39.91 (37.59, 46.63)	0.8640	0.4582	0.1737	0.1882
TEWL	6.85 (4.78, 8.75)	8.9 (5.5, 11.6)	8.35 (4.77, 11.22)	6.85 (4.25, 12.1)	0.4932	0.2702	0.3792	0.6849
Melanin	188.46 (140, 223.25)	146.83 (128, 191.08)	163.29 (135.09, 218.17)	144 (112.54, 185.66)	0.0063^*^	0.0006†	0.7694	0.3569
Erythema	299.84 (263.84, 357.55)	291.5 (255.41, 325.5)	250.83 (170.92, 317.08)	241.84 (199.29, 296.17)	0.3803	0.7084	0.0892	0.0941
L	65.48 (63.19, 68.51)	66.62 (63.25, 68.5)	66.25 (59.75, 68.83)	66.98 (64.01, 69.37)	0.9134	0.0197^*^	0.7075	0.6588
A	12.84 (10.88, 13.74)	12.28 (11.28, 12.98)	11.26 (9.9, 13.44)	10.96 (9.91, 12.04)	0.6813	0.3378	0.4238	0.0787
B	12.03 (10.61, 13.16)	11.95 (10.49, 13.5)	13.73 (12.09, 15.38)	12.44 (10.98, 13.55)	0.5289	0.0427^*^	0.0223^*^	0.3483
ITA	53.16 (43.83, 58.42)	52.67 (48.5, 57.71)	50.34 (36.42, 55.75)	52.84 (45.17, 58.5)	0.8590	0.0045^**^	0.2187	0.9892
Ra	21.75 (19, 24.38)	19.25 (17.75, 22.75)	20.5 (19, 22.75)	20 (19, 23.25)	0.0002†	0.3075	0.7449	0.3356
Rmax	153.25 (123.75, 175)	139 (120.88, 157.5)	133 (121.25, 161.25)	141.5 (126.75, 155.75)	0.0002†	0.8959	0.5338	0.5323
R0	0.12 (0.08, 0.2)	0.12 (0.06, 0.19)	0.20 (0.13, 0.27)	0.12 (0.08, 0.24)	0.3424	0.0076^*^	0.0228^*^	0.2447
R2	0.75 (0.68, 0.84)	0.83 (0.62, 0.86)	0.79 (0.73, 0.83)	0.80 (0.71, 0.83)	0.4222	0.2984	0.1969	0.4488
R5	0.47 (0.4, 0.55)	0.51 (0.32, 0.57)	0.66 (0.56, 0.76)	0.63 (0.57, 0.69)	0.8698	0.3225	0.0042^*^	0.0116^*^

Comparisons between time points (i.e., St_0_ vs. St_25_ and HCSt_0_ vs. HCSt_25_) were performed by the Student's *t*-test for paired samples or by the Wilcoxon test for paired samples; comparisons between treatments at the same time point were performed by the Student's *t*-test for unpaired samples or by the Wilcoxon rank-sum test for unpaired samples (i.e., St_0_ vs. HCSt_0_ and St_25_ vs. HCSt_25_).

St_0_ = median (25^th^, 75^th^ percentiles, IQR) of each variable's distribution in scars at t_0_.

St_25_ = median (25^th^, 75^th^ percentiles, IQR) of each variable's distribution in scars at t_25_.

HCSt_0_ = median (25^th^, 75^th^ percentiles, IQR) of each variable's distribution in healthy contralateral skin sites at t_0_.

HCSt_25_ = median (25^th^, 75^th^ percentiles, IQR) of each variable's distribution in healthy contralateral skin sites at t_25_.

St_0_ versus St_25_ = *p* value deriving from the comparison of each variable's distribution between t_0_ and t_25_ measurements in scars.

HCSt_0_ versus HCSt_25_ = *p* value deriving from the comparison of each variable's distribution between t_0_ and t_25_ measurements in healthy contralateral skin sites.

St_0_ versus HCSt_0_ = *p* value deriving from the comparison of each variable's distribution between measurements in scars and healthy contralateral skin sites at t_0_.

St_25_ versus HCSt_25_ = *p* value deriving from the comparison of each variable's distribution between measurements in scars and healthy contralateral skin sites at t_25_.

Variable = analyzed variable.

HCS, healthy contralateral skin sites; IQR, interquartile range; S, scars; TEWL, transepidermal water loss.

^*^ *p* Value <0.05.

†*p* Value <0.004 based on the Bonferroni correction for multiple testing.

### Corneometry

The *t*-test for paired samples failed to demonstrate any statistically significant difference in the scars before (t_0_) and after the treatment (t_25_).

### Transepidermal water loss

The *t*-test for paired samples failed to demonstrate any statistically significant difference in the scars before (t_0_) and after the treatment (t_25_). The healthy contralateral skin displayed no changes at the same times.

### Elastometry

#### R0

The *t*-test for paired samples failed to demonstrate any statistically significant difference in the scars before (t_0_) and after the treatment (t_25_).

A statistically significant difference was appreciated in the healthy contralateral skin at the same times (*p* = 0.0076).

The scars showed a statistically significant lower R0 index versus the healthy contralateral skin before the treatment (t_0_), while no difference was appreciated at the end of the treatment (*p* = 0.0228).

#### R2

The Wilcoxon test for paired samples failed to demonstrate any statistically significant difference in the scars before (t_0_) and after the treatment (t_25_).

#### R5

The *t*-test for paired samples failed to demonstrate any statistically significant difference both in the scars and in the healthy contralateral skin before (t_0_) and after the treatment (t_25_). A statistically significant difference was demonstrated between the scar and the healthy contralateral skin before the treatment (*p* = 0.0042); such a difference persisted after the treatment, although with a lesser degree (*p* = 0.0116).

### Colorimetry

#### Mexameter method

##### Melanin

A statistically significant reduction in the MI was appreciated in the scars after the treatment (*p* = 0.0063) and in the healthy contralateral skin at the same time (*p* = 0.0006). No statistically significant difference was appreciated in the scars versus the healthy contralateral skin both before and after the treatment.

##### Erythema

The *t*-test for paired samples failed to demonstrate any statistically significant difference both in the scars and the healthy contralateral skin before (t_0_) and after the treatment (t_25_).

#### Colorimeter method

*L:* the *t*-test for paired samples failed to demonstrate any statistically significant difference in the scars before (t_0_) and after the treatment (t_25_), while a statistically significant increase in the L index was appreciated in the healthy contralateral skin after the treatment (t_25_; *p* = 0.0197).

*A:* the *t*-test for paired samples failed to demonstrate any statistically significant difference in the scars before (t_0_) and after the treatment (t_25_), although a trend was appreciated in the A index that approached the healthy contralateral skin, displaying a drift toward the green section of the light spectrum. No changes were appreciated in the healthy contralateral skin before (t_0_) and after the treatment (t_25_).

*B:* the *t*-test for paired samples failed to demonstrate any statistically significant difference in the scars before (t_0_) and after the treatment (t_25_), although a trend was appreciated in the B index that approached the healthy contralateral skin as demonstrated by the loss of statistically significant difference between the scars and the healthy contralateral skin after the treatment with a drift toward the blue section of the light spectrum. A statistically significant difference was appreciated in the healthy contralateral skin before (t_0_) and after the treatment (t_25_), displaying a drift toward the blue section of the light spectrum too (*p* = 0.0427).

*ITA:* the *t*-test for paired samples failed to demonstrate any statistically significant difference in the scars before (t_0_) and after the treatment (t_25_). A statistically significant difference was appreciated in the healthy contralateral skin at the same times (*p* = 0.0045).

### 3D skin scan

#### Ra

A statistically significant difference (*p* = 0.0002) was appreciated in the scars before (t_0_) and after the treatment (t_25_), with a lower Ra index after the treatment. No changes were appreciated in the healthy contralateral skin.

#### Rmax

A statistically significant difference (*p* = 0.0002) was appreciated in the scars before (t_0_) and after the treatment (t_25_) with the scar tending to approach the wrinkling of the normal skin. No changes were appreciated in the healthy contralateral skin.

### Impact of confounding factors

The impact of potential confounders on the statistically significant variations identified was also tested. The individual-level variations observed by comparing before (t_0_) versus after the treatment (t_25_) measurements showed evidence of weak correlation with scar age (*r*^2^ < 0.05) and did not vary significantly among aethiology classes (*p* > 0.05). These observations suggest that the identified variations were not influenced by the effect of confounding factors.

### VAS and PSAS questionnaires

The results from the subjective assessments are summarized in Table 3.

**Table 3.** Variables Distribution of VAS and PSAS Questionnaires at Different Time Points and Corresponding Variations

				Δ *(t_16_–t_0_)*		Δ *(t_25_–t_16_)*		Δ *(t_25_–t_0_)*	
*Variable*	*t_0_ Median (IQR)*	*t_16_ Median (IQR)*	*t_25_ Median (IQR)*	*Median (IQR)*	p	*Median (IQR)*	p	*Median (IQR)*	p
VAS researcher	6 (5, 8)	4 (3, 6)	3 (2, 4)	−2 (−2, −1.5)	0.0002^*^	−1 (−1.62, −0.88)	0.0007^*^	−3 (−3.62, −2)	2.52 × 10^−09^^*^
VAS patient	7.25 (6, 8)	5 (2.38, 6.25)	3 (1.75, 5)	−2 (−3.25, −1.5)	2.52 × 10^−06^^*^	−1 (−2, −0.5)	6.06 × 10^−05^^*^	−3.5 (−5, −2.5)	3.18 × 10^−08^^*^
PSAS	20 (16.75, 23.25)	11.5 (7, 14)	9.5 (6.5, 12.25)	−7.5 (−10.25, −6)	9.45 × 10^−08^^*^	−2 (−4, −1.5)	2.31 × 10^−05^^*^	−11 (−13, −6.75)	2.12 × 10^−10^^*^

Median (IQR) = median value (25^th^, 75^th^ percentiles) of each variable's distribution;

*p* value = *p* value from the Student's *t*-test for paired samples [variables following the normal distribution (Shapiro test *p* ≥ 0.05)] or from the Wilcoxon Signed Rank test for paired samples [variables deviating from the normal distribution (Shapiro test *p* < 0.05)], comparing variables values between different time points.

IQR, interquartile range.

^*^ *p* < 0.05.

The VAS and PSAS questionnaires demonstrated a statistically significant improvement in all of the items in the pretreatment (t_0_) versus the post-treatment time (t_25_). A similar statistically significant improvement was also appreciated in the pretreatment versus midtreatment time (t_16_).

## Discussion

**FIG. 3.**
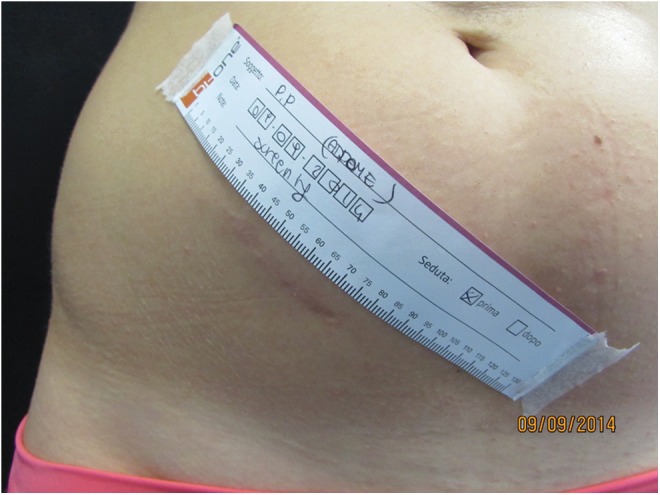
Mature scar in the right iliac fossa. Pretreatment view.

**FIG. 4.**
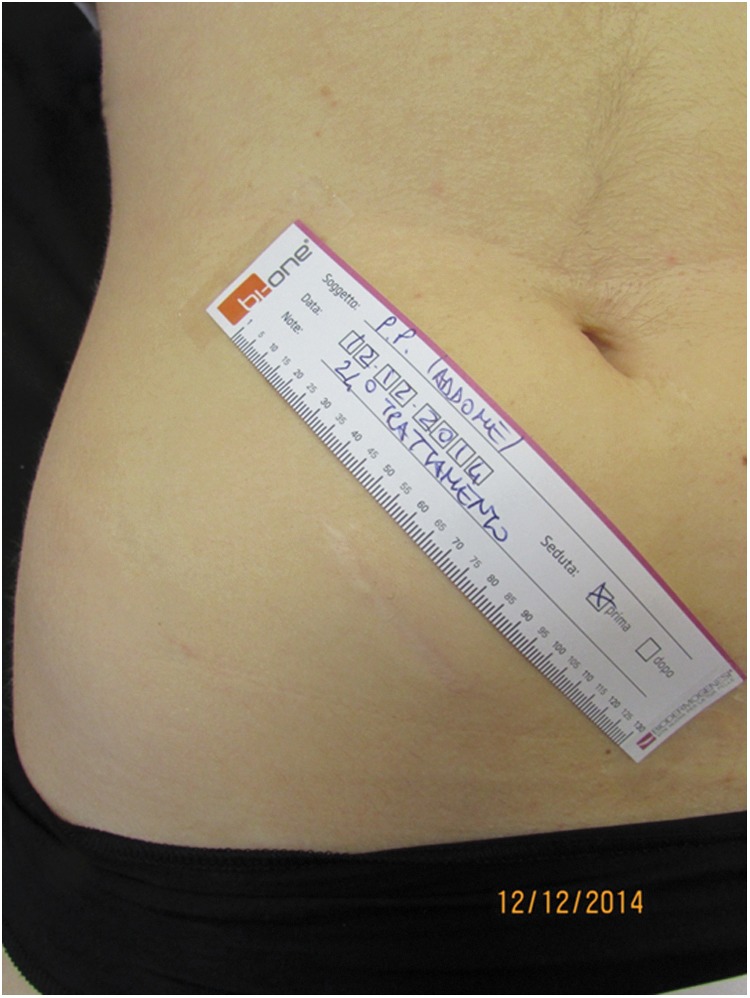
Post-treatment view with overall scar improvement.

Our results demonstrated an overall fair improvement in the scar connective tissue after the combined sequential local treatment with low-intensity electromagnetic and electric stimulation in association with negative pressure (Figs. 3 and 4) as demonstrated by the changes in the following objectively assessed anatomical functional parameters: net skin elasticity (R5) expressed as the ratio between the maximum skin extension and the residual skin deformity, blue and yellow light reflection (B), skin surface profile (Ra), and the difference between the highest skin surface spot and the deepest skin furrow (Rmax).

The final assessment was carried out shortly after the last application. Such a schedule was considered the best compromise to meet both the patients' compliance and the onset of biological effects induced by the sequential treatments, as the ultrastructural changes in the collagen fibril architecture are demonstrated to occur shortly after the radiofrequency application.^5,20^ The patients were advised to contact the research staff in case of unfavorable mid and long-term scar evolution after the treatments, but no complaints were referred.

Although some degree of elastic fiber network regeneration has been reported in mature scars, the latter are demonstrated to be still remarkably stiffer than the normal skin.^3^

Skin compactness (R0) did not demonstrate any change in the scars after the treatment, although the healthy contralateral skin underwent a decrease in such an index at the same time, likely due to a seasonal change, not influent on the scar tissue. No changes were appreciated in skin resistance (R2), both in the scar and in the contralateral healthy skin. However, the lesser significance in the difference of net skin elasticity (R5) between the scars and the contralateral healthy skin at the end of the treatment might suggest a slight improvement of the overall scar elasticity.

Elastic fiber regeneration has been reported following radiofrequency treatments^21^ with evidence of a juvenile reticular pattern.^22^ Therefore, our results are likely to be related to the radiofrequency sequential applications.

In our sample, the significant similar decrease of the MI in the scars and in the healthy contralateral skin is likely to be related to seasonal regression of skin tanning.

Skin owes its color to four pigments:^23^ oxyhemoglobin, reduced hemoglobin, melanin, and carotene. Blue color is an optical effect due to a Tyndall phenomenon as light reflected *in vivo* from the melanin, according to an exponential dependence on wavelength,^24^ is scattered by the turbid medium of the basal epidermis.

Evidence has been produced to suggest that the overlying epithelium contributes nothing to the blue colors, and it has been proposed that the brown pigment in the corium appears blue because of subtractive mixing of colors.^25^ Collagen reflects blue by withdrawal of longer wavelengths of light spectrum.^25–27^

In our sample, the colorimetry displayed some interesting outcomes in the scars after the treatment.

**FIG. 5.**
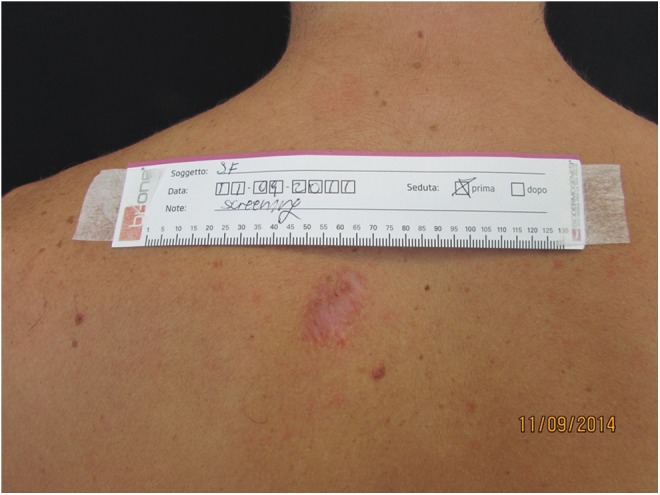
Mature scar in the dorsum. Pretreatment view.

**FIG. 6.**
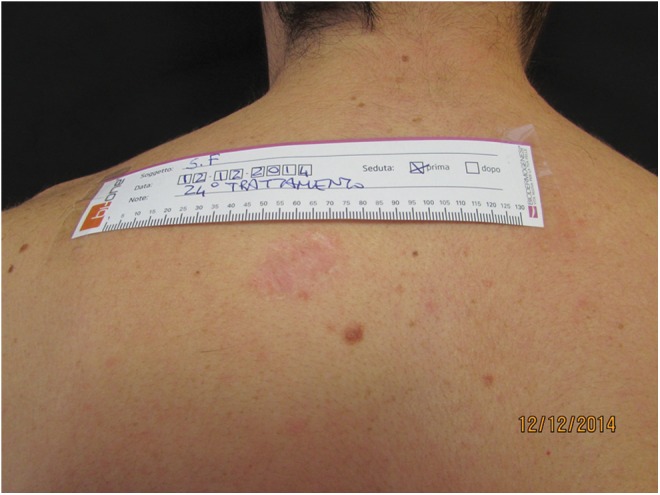
Post-treatment view with improved scar thickness and color.

The B-index showed an interesting trend, although no statistically significant difference was appreciated in the scars after the treatment; their B-index tended to approach that of the normal skin at the end of the applications, thus suggesting that the scars tended to drift toward the blue section of the light spectrum and to approach the color of healthy skin (Figs. 5 and 6).

The A-index in the scars displayed a drift toward the green section of the light spectrum after the treatment, thus approaching that of the healthy contralateral skin too.

The absence of any significant effect of the treatment on both melanin and erythema indexes suggests that the drift toward the cold section of the light spectrum might be related to collagen fiber rearrangement.

The 3D synthetic assessment of the skin surface, expressed by the Ra and Rmax indexes, demonstrated that the surface wrinkling was more represented in the scars versus the healthy contralateral skin before the treatment. A significant improvement with a lower Ra and Rmax indexes in the scars versus the healthy contralateral skin was demonstrated at the end of the treatment. The treatment, therefore, yielded both a reduction of the scar surface wrinkling and an overall scar flattening.

All of the latter changes observed in the assessed anatomical functional parameters are likely to be related to the alterations of the scar collagen structure induced by the treatment.

The favorable objective results consistently matched the outcomes from the VAS and PSAS questionnaires. Actually the VAS Scale demonstrated a significant overall improvement in the scar perception both in the patients and in the medical researcher; according to the PSAS Scale, the patients referred a significant reduction of scar-related pain, stiffness, thickness, and color after the treatment.

**FIG. 7.**
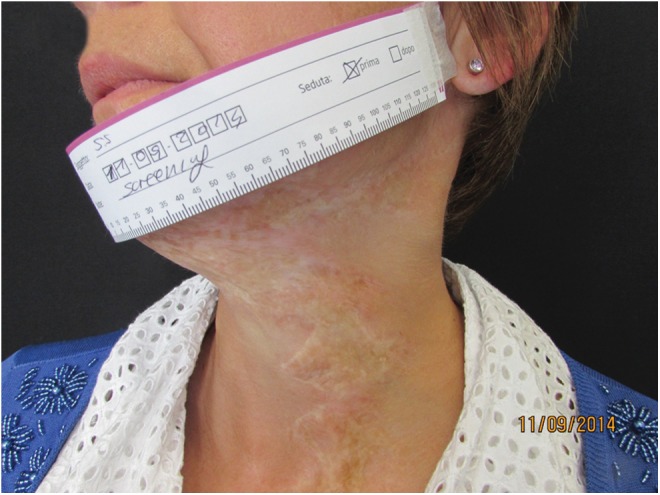
Mature scar in the left side of the neck. Pretreatment view.

**FIG. 8.**
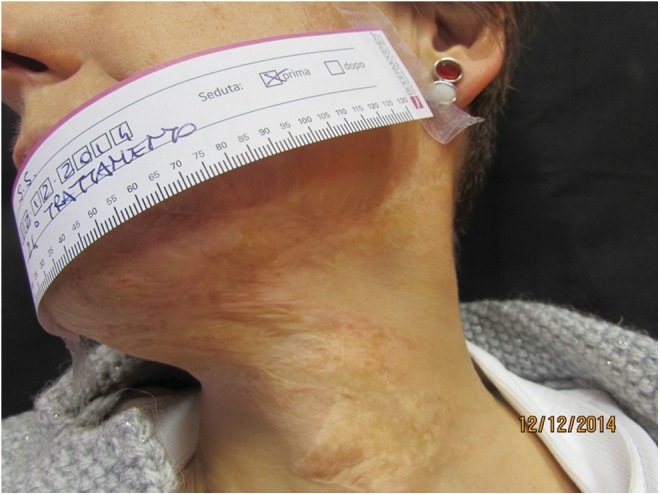
Post-treatment view with scar elasticity improvement.

All of the modifications observed in the scars after the treatment are likely to be related to the peculiar action of radiofrequency, which allows selective heat transfer to the dermis and subcutaneous tissue, yielding a controlled collagen alteration. Several literature reports^5,22,28,29^ demonstrated that radiofrequencies provide an immediate heat-induced rearrangement of native collagen fibers that are gently progressively denaturated and progressively metabolized by the macrophages and that a late fibroblastic response yields regeneration of the normal dermal collagen and elastic fiber network. These effects might, therefore, explain the reduction of the scar surface wrinkling, the overall scar flattening, the changes in the skin light absorption properties, and the favorable trend of slight net scar elasticity increase after the treatment in our sample (Figs. 7 and 8).

Actually, the radiofrequency-related changes in the connective tissue are not entirely explained by a simple temperature rise. It is demonstrated that collagen fibers begin to curve at 52°C–55°C,^30^ contract at 65°C,^31^ and the denaturation threshold falls between 60°C and 70°C.^32^ As the maximum power output in our device provides a the temperature range of 39°C–40°C, the supposed changes of the collagen and elastic fibers might also be related to a spatial rearrangement in the absence of complete denaturation.^22^

The absence of any significant effect on corneometry, transepidermal water loss, and MI confirms that radiofrequencies within the therapeutic range power selectively act on the dermis and spare the epidermis.^22^ Therefore, the procedure under study can be considered safe without side effects on epithelia and melanocytes.

The applied negative pressure improves the radiofrequency effects mainly providing a dermal neoangiogenesis that, in turn, supports all of the regenerative processes.^33–35^

A further improvement of the effects of the combined application of radiofrequency and negative pressure is also related to the square wave electric pulses whose favorable effects on the cells' activity have been long demonstrated.

The favorable effects of monopolar capacitive radiofrequencies have been long investigated in the orthopedic pathology where they are widely used in therapy-related clinical practice for their thermal effects, mainly relieving pain and inflammation and improving tissue extensibility. Even if the most commonly used and researched are shortwave therapies, new electrophysical agents employing much lower frequencies have emerged, but, to date, they remain largely unexplored.^36–39^

Our experience, therefore, would bring new knowledge about the use of capacitive radiofrequency into new fields of clinical application.

The objective assessment of scars is a difficult matter as the recruitment of an actual homogeneous sample is hampered by the extreme variability of the individual response of the wound healing process, which is only partially related to the scar aethiology and clinical features.^40^

Recruiting a homogeneous sample of scars from patients with a high degree of personal compliance is even more difficult. Such pitfalls undoubtedly represent a limitation in this trial. Nevertheless, the results from this study might suggest further investigations in the field of the interactions between the physical energies and the living tissues and might contribute to increase the treatment options for scars.

## Conclusions

The combined sequential local treatment of mature scars with low-intensity monopolar capacitive radiofrequency and electric stimulation in association with negative pressure objectively demonstrated an overall favorable synergic effect in the scar connective tissue.

Collagen fiber rearrangement likely yielded to a change in the color features with a drift toward the cold section of the light spectrum and to a reduction of the surface wrinkling with an overall flattening.

Net skin elasticity slightly improved too, likely following elastic fiber network remodeling.

These objective results matched the favorable outcomes from the subjective VAS and PSAS questionnaires.

No side effects on epithelia and melanocytes were appreciated, thus demonstrating a selective action of the treatment on the dermis without involving the epidermis.

Therefore, the favorable results observed in our study on mature scars might be related to a synergic effect of three different well-known physical energies currently used in clinical practice.
